# Supplementary material for: SARS-CoV-2 antibodies recognize 23 distinct epitopic sites on the receptor binding domain
Source: Commun Biol. 2023 Sep 19;6:953. doi: 10.1038/s42003-023-05332-w (PMC10509263; doi:10.1038/s42003-023-05332-w)
Supplement: Supplementary file 2 — Supplementary Material [file 42003_2023_5332_MOESM2_ESM.pdf]

Supplementary Figures and Tables for:

SARS-CoV-2 antibodies recognize 23 distinct epitopic sites on the receptor binding domain

Jiansheng Jiang, Christopher T. Boughter, Javeed Ahmad, Kannan Natarajan, Lisa F. Boyd, Martin Meier-Schellersheim, David H. Margulies

This file contains in order:

Supplementary Table 1

Supplementary Fig. 1

Supplementary Table 2a, 2b and 2c

Supplementary Fig. 3a, 3b, 3c and 3d

Supplementary Table 3a and 3b

Supplementary Table 4a and 4b

Supplementary Fig. 4

Supplementary Fig. 5

Supplementary Fig. 6

Supplementary Fig. 7

References to Supplementary Figures and Tables

Supplementary Table 1

Barnes	Definition
**Class 1**	Block ACE2 and bind “up” RBD only
**Class 2**	Block ACE2 and bind “up” and “down” RBD
**Class 3**	Fail to block ACE2 but bind “up” & “down” RBD
**Class 4**	Fail to block ACE2 but bind “up” RBD only

Supplementary Table 1. Characteristics of Barnes classification of Ab to the RBD of SARS-CoV-2. As described by Barnes et al [1], antibodies are classified by the location of their epitopes, in either the “up” and/or “down” orientations of the RBD in the spike trimer, and also on their ability to block binding to the cellular receptor, ACE2.

Supplementary Fig. 1

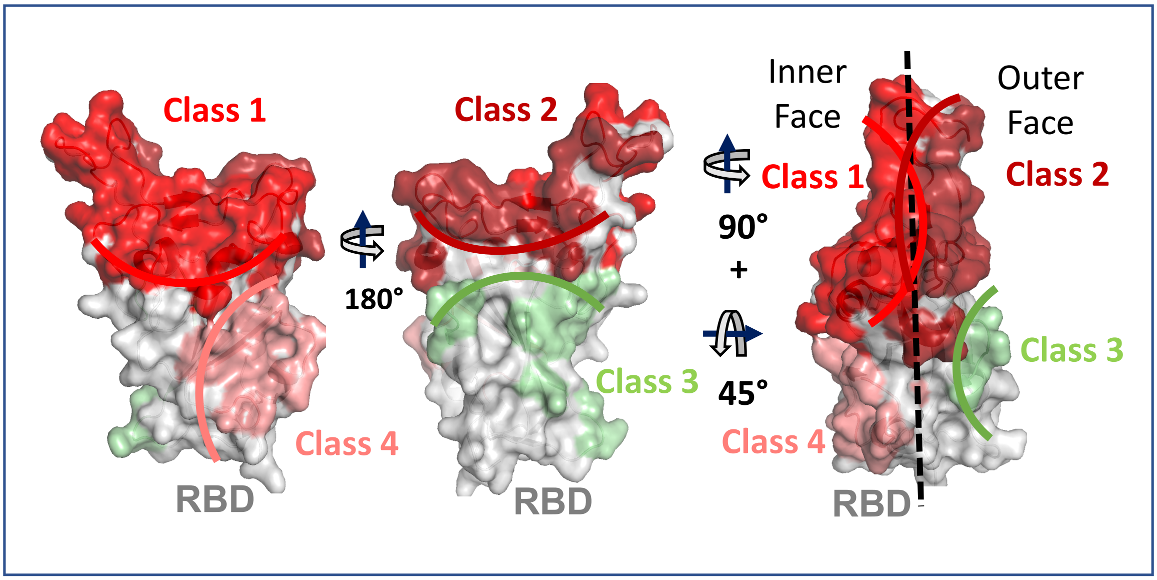

Supplementary Fig. 1. Graphic depiction (footprint) of Barnes classification [1]. Surface representation of the SARS-CoV-2 RBD (PDB 7BZ5_A) is displayed with epitopic regions colored: Class 1, red; Class 2, firebrick; Class 3, lime; Class 4, salmon. There are many overlaps between Class 1 and Class 2.

Supplementary Fig. 2

a

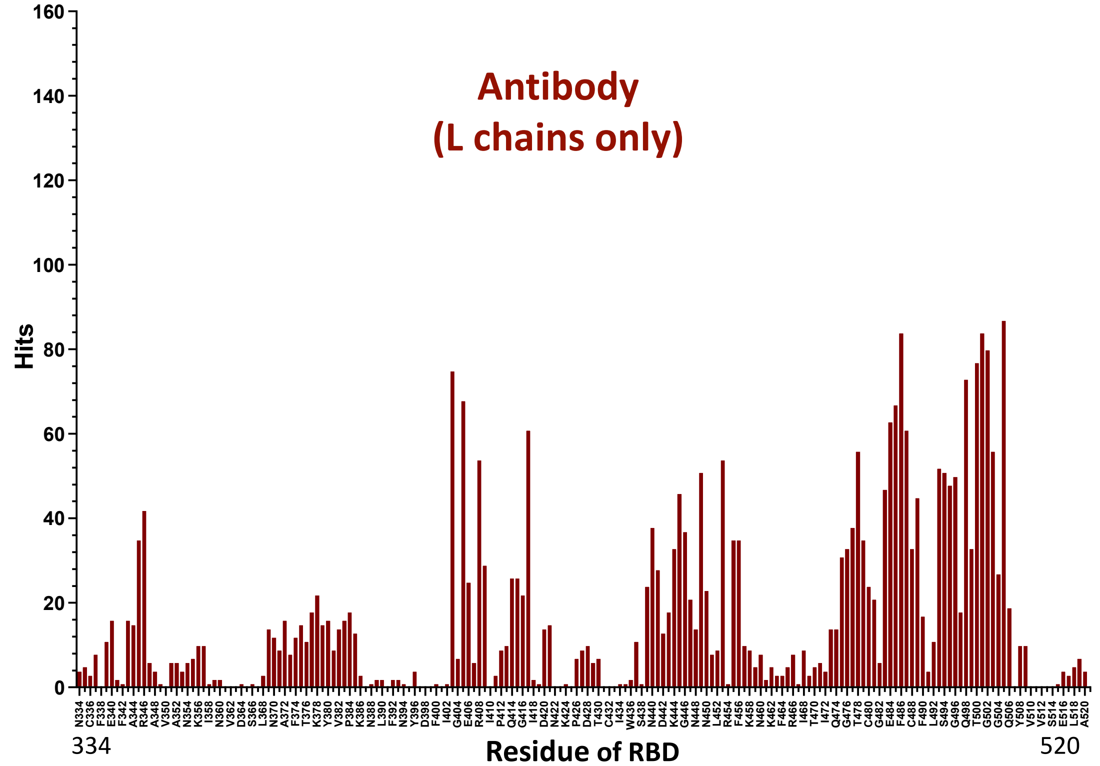

b

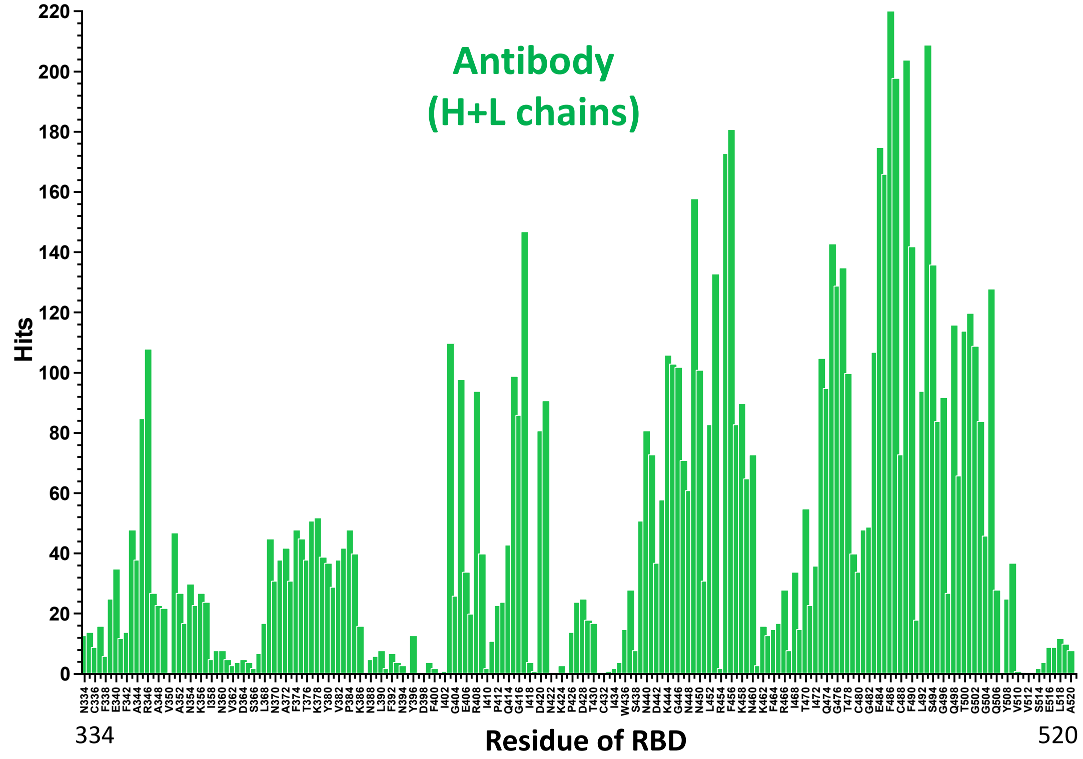

Supplementary Fig. 2. Number of contacts to RBD by Ab L chains. Total number of contacts to each of the indicated RBD residues summed from all available Ab X-ray and cryo-EM structures for Ab L chains. The odd number RBD residue names are omitted for clarity.

Supplementary Table 2

a

ES	RANGE	RBD	Hits	(%)	NONCDR	(%)	CDR1	(%)	CDR2	(%)	CDR3	(%)
0	-------	--------	226	4.02	15	0.27	25	0.44	49	0.87	137	2.44
1	339-341	GEV	44	0.78	11	0.20	4	0.07	5	0.09	24	0.43
2	343-349	NTRFAS	229	4.07	27	0.48	17	0.30	58	1.03	127	2.26
3	351-357	YAWNRKR	146	2.60	23	0.41	14	0.25	33	0.59	76	1.35
4	368-374	LYNSASF	178	3.17	14	0.25	9	0.16	35	0.62	120	2.13
5	375-380	STFKCY	163	2.90	22	0.39	10	0.18	16	0.28	115	2.05
6	381-386	GVSPTK	140	2.49	20	0.36	22	0.39	16	0.28	82	1.46
7	403-409	RGDEVRQ	155	2.76	24	0.43	18	0.32	20	0.36	93	1.65
8	411-417	APGQTGK	276	4.91	121	2.15	29	0.52	56	1.00	70	1.24
9	420-428	DYNYKLPDD	182	3.24	15	0.27	21	0.37	116	2.06	30	0.53
10	437-443	NSNNLDS	202	3.59	19	0.34	14	0.25	32	0.57	137	2.44
11	444-449	KVGGNY	399	7.10	125	2.22	68	1.21	88	1.57	118	2.10
12	450-454	NYLYR	257	4.57	33	0.59	32	0.57	60	1.07	132	2.35
13	455-460	LFRKSN	561	9.98	101	1.80	66	1.17	226	4.02	168	2.99
14	462-467	KPFERD	72	1.28	17	0.30	9	0.16	10	0.18	36	0.64
15	468-472	ISTEI	137	2.44	34	0.60	25	0.44	30	0.53	48	0.85
16	473-479	YQAGSTP	525	9.34	41	0.73	299	5.32	7	0.12	178	3.17
17	481-484	NGVE	245	4.36	103	1.83	15	0.27	59	1.05	68	1.21
18	485-487	GFN	397	7.06	90	1.60	44	0.78	54	0.96	209	3.72
19	488-491	CYFP	336	5.98	40	0.71	28	0.50	71	1.26	197	3.50
20	492-496	LQSYG	402	7.15	25	0.44	88	1.57	93	1.65	196	3.49
21	497-502	FQPTNG	190	3.38	48	0.85	46	0.82	25	0.44	71	1.26
22	503-509	VGYQPYR	136	2.42	23	0.41	17	0.30	17	0.30	79	1.40
23	516-520	ELLHA	25	0.44	5	0.09	4	0.07	0	0.00	16	0.28
SUM	--------	--------	5623	100.00	996	17.71	924	16.43	1176	20.91	2527	44.94

b

ES	RANGE	RBD	Hits	(%)	NONCDR	(%)	CDR1	(%)	CDR2	(%)	CDR3	(%)
0	-------	--------	102	3.28	21	0.68	52	1.67	12	0.39	17	0.55
1	339-341	GEV	29	0.93	9	0.29	9	0.29	2	0.06	9	0.29
2	343-349	NTRFAS	114	3.67	40	1.29	39	1.26	8	0.26	27	0.87
3	351-357	YAWNRKR	49	1.58	14	0.45	25	0.80	6	0.19	4	0.13
4	368-374	LYNSASF	74	2.38	17	0.55	34	1.09	10	0.32	13	0.42
5	375-380	STFKCY	97	3.12	21	0.68	30	0.97	17	0.55	29	0.93
6	381-386	GVSPTK	73	2.35	22	0.71	20	0.64	12	0.39	19	0.61
7	403-409	RGDEVRQ	264	8.50	61	1.96	41	1.32	28	0.90	134	4.31
8	411-417	APGQTGK	157	5.05	53	1.71	30	0.97	37	1.19	37	1.19
9	420-428	DYNYKLPDD	56	1.80	21	0.68	15	0.48	14	0.45	6	0.19
10	437-443	NSNNLDS	133	4.28	30	0.97	67	2.16	9	0.29	27	0.87
11	444-449	KVGGNY	202	6.50	77	2.48	39	1.26	28	0.90	58	1.87
12	450-454	NYLYR	95	3.06	14	0.45	54	1.74	7	0.23	20	0.64
13	455-460	LFRKSN	102	3.28	19	0.61	42	1.35	29	0.93	12	0.39
14	462-467	KPFERD	25	0.80	5	0.16	12	0.39	5	0.16	3	0.10
15	468-472	ISTEI	27	0.87	9	0.29	7	0.23	5	0.16	6	0.19
16	473-479	YQAGSTP	221	7.11	51	1.64	125	4.02	4	0.13	41	1.32
17	481-484	NGVE	138	4.44	40	1.29	36	1.16	13	0.42	49	1.58
18	485-487	GFN	241	7.76	76	2.45	59	1.90	14	0.45	92	2.96
19	488-491	CYFP	99	3.19	30	0.97	33	1.06	8	0.26	28	0.90
20	492-496	LQSYG	212	6.82	18	0.58	166	5.34	10	0.32	18	0.58
21	497-502	FQPTNG	365	11.75	73	2.35	232	7.47	8	0.26	52	1.67
22	503-509	VGYQPYR	209	6.73	45	1.45	83	2.67	13	0.42	68	2.19
23	516-520	ELLHA	23	0.74	6	0.19	13	0.42	3	0.10	1	0.03
SUM	-------	--------	3107	100	772	24.85	1263	40.65	302	9.72	770	24.78

c

ES	RANGE	RBD	Hits	(%)	NONCDR	(%)	CDR1	(%)	CDR2	(%)	CDR3	(%)
0	-------	--------	101	5.50	28	1.53	12	0.65	10	0.54	51	2.78
1	339-341	GEV	3	0.16	0	0.00	0	0.00	0	0.00	3	0.16
2	343-349	NTRFAS	52	2.83	12	0.65	7	0.38	0	0.00	33	1.80
3	351-357	YAWNRKR	69	3.76	19	1.03	6	0.33	9	0.49	35	1.91
4	368-374	LYNSASF	129	7.03	18	0.98	0	0.00	24	1.31	87	4.74
5	375-380	STFKCY	128	6.97	29	1.58	5	0.27	4	0.22	90	4.90
6	381-386	GVSPTK	101	5.50	12	0.65	26	1.42	12	0.65	51	2.78
7	403-409	RGDEVRQ	86	4.68	10	0.54	7	0.38	3	0.16	66	3.59
8	411-417	APGQTGK	51	2.78	19	1.03	7	0.38	3	0.16	22	1.20
9	420-428	DYNYKLPDD	31	1.69	10	0.54	6	0.33	1	0.05	14	0.76
10	437-443	NSNNLDS	27	1.47	19	1.03	0	0.00	1	0.05	7	0.38
11	444-449	KVGGNY	154	8.39	68	3.70	32	1.74	31	1.69	23	1.25
12	450-454	NYLYR	105	5.72	24	1.31	22	1.20	22	1.20	37	2.02
13	455-460	LFRKSN	78	4.25	13	0.71	7	0.38	3	0.16	55	3.00
14	462-467	KPFERD	24	1.31	6	0.33	3	0.16	0	0.00	15	0.82
15	468-472	ISTEI	82	4.47	29	1.58	9	0.49	29	1.58	15	0.82
16	473-479	YQAGSTP	13	0.71	2	0.11	2	0.11	0	0.00	9	0.49
17	481-484	NGVE	97	5.28	30	1.63	9	0.49	44	2.40	14	0.76
18	485-487	GFN	67	3.65	24	1.31	3	0.16	15	0.82	25	1.36
19	488-491	CYFP	108	5.88	22	1.20	12	0.65	11	0.60	63	3.43
20	492-496	LQSYG	178	9.69	45	2.45	39	2.12	21	1.14	73	3.98
21	497-502	FQPTNG	54	2.94	25	1.36	12	0.65	10	0.54	7	0.38
22	503-509	VGYQPYR	76	4.14	22	1.20	4	0.22	4	0.22	46	2.51
23	516-520	ELLHA	22	1.20	7	0.38	2	0.11	2	0.11	11	0.60
SUM	-------	--------	1836	100	493	26.85	232	12.64	259	14.11	852	46.41

Supplementary Table 2. **Statistics for CDR loop distribution (contact numbers and percentage) on 23 ES**. **a** Antibody Heavy chains. **b** Antibody Light chains. **c** Nanobodies.

Supplementary Fig. 3

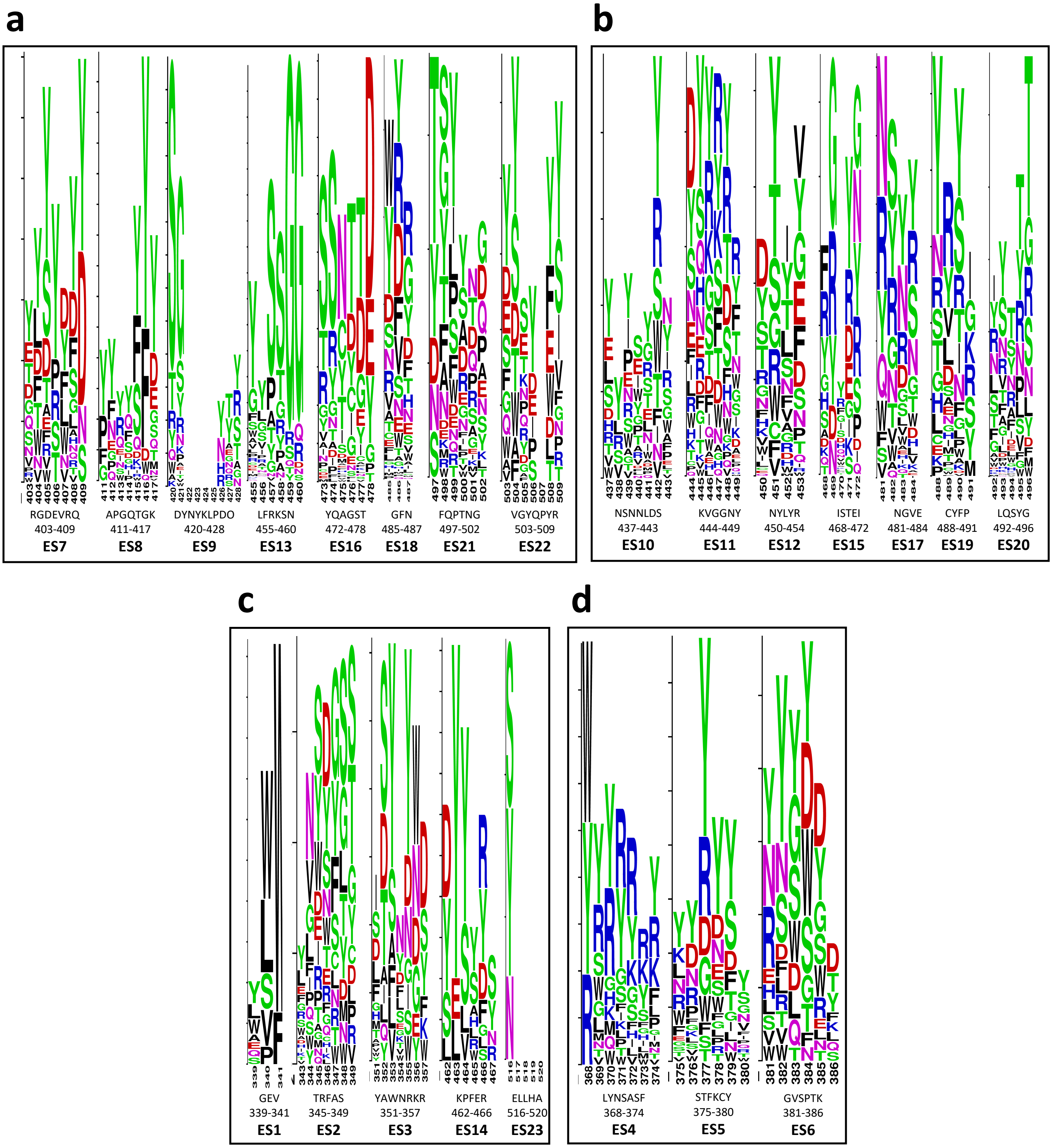

Supplementary Fig. 3 Paratope (antibody H chain) amino acids against 23 epitopic sites (ES) on RBD. WebLogo plots of antibody (H chain) sequences in contact with each residues of RBD. The plots are grouped against each ES. The size of letter (logo) reflects relative frequency of the amino acids within the group [2]). **a** Class-1, ES=(7,8,9,13,16,18,21,22), **b** Class-2, ES=(10,11,12,15,17,19,20), **c** Class-3, ES=(1,2,3,14,23), **d** Class-4, ES=(4,5,6).

Supplementary Table 3a

CLUSTER	MEMBERS	ES	LIST (Similarity>=0.85)
A1	56	(8,9,12,13,16,17,18,19,20)	PDI-231,CV2-2325,A8-1,COVA2-04,Windsor-C98C7,P5A-3C8,CB6,C1A-B12,C102,BG4-25,BD-629,BD-604,P5A-2F11,AZ090,PDI-42,BD-508,BD-503,TAU-2303,scFv-E4,R40-1G8,PDI-37,PDI-222,P4A1,P2C-1F11,P22A-1D1,Omi-3,Omi-18,Minenkova-76,ION-360,CV30,COVOX-40,COVOX-269,COVOX-253,COVOX-222,COVOX-150,CoV11,CC12-3,C099,C098,Beta-27,BD-744,BD-236,ab1,WRAIR-2125,UT28K,CV503,Windsor-G32A4,COV2-2196,STE90-C11,P5A-1B8,LY-CoV481,FI-3A,B38,A19-61-1,910-30,10D12-ab1,COVOX-158
A2	45	(8,9,13,15,16,18,19,20)	nCoV-61,TAU-2303,scFv-E4,R40-1G8,PDI-37,PDI-222,P4A1,P2C-1F11,P22A-1D1,Omi-3,Omi-18,Minenkova-76,ION-360,CV30,COVOX-40,COVOX-269,COVOX-253,COVOX-222,COVOX-150,CoV11,CC12-3,C099,C098,Beta-27,BD-744,BD-236,ab1,P2B-1A10,STE90-C11,P5A-1B8,LY-CoV481,FI-3A,B38,A19-61-1,910-30,10D12-ab1,ZCB11,XGv265,Omi-12,Ab-58G6,CV2-1169,B1-182-1,A23-58-1,P5A-3A1,LY-CoV488,BD-515
A3	39	(7,8,9,12,13,16,18,19,20,22)	P5A-1D2,C1A-F10,C1A-C2,Beta-47,PDI-96,Windsor-C98C7,P5A-3C8,CB6,C1A-B12,C102,BG4-25,BD-629,BD-604,TAU-2303,scFv-E4,R40-1G8,PDI-37,PDI-222,P4A1,P2C-1F11,P22A-1D1,Omi-3,Omi-18,Minenkova-76,ION-360,CV30,COVOX-40,COVOX-269,COVOX-253,COVOX-222,COVOX-150,CoV11,CC12-3,C099,C098,Beta-27,BD-744,BD-236,ab1,C105
A4	21	(11,12,13,17,18,19,20,21)	S2-M11,P5A-1B9,P2C-1A3,Fab2-4,Fab2-15,C121,2B04,TAU-2212,CA521,nCov-617,COVOX-384,C002,Fab2-43,COVOX-316,Ab496,Ab354,2H2,Omi-25,Fab-15033-7,Fab-15033,WRAIR-2173,P2B4
A5	19	(2,10,11,12,21,22)	S2X324,M8a-28,LY-CoV1404,C032,BD55-5840,BD55-3546,BD-812,32C7-Fab30,XG005,X10,JS026,FD-11A,A5-10,2H04,PR961,hu33,Fab2-7,COV2-2130,BD-804,AZD-1061
A6	18	(2,3,11,12,13,14,15,17,19,20)	R1-32,PDI-215,Li-D2,COVOX-58,BD-813,6M6,35B5,BD-667,Ab326,Zhou-47D1,PDI-93,P2B-2F6,P1D9,Omi-31,NA8,N-612-017,Fab1-57,Fab-52,COVOX-278
A7	16	(2,3,11,12,15,17,19,20,21)	Omi-6,BD-667,CV07-270,Zhou-47D1,PDI-93,P2B-2F6,P1D9,Omi-31,NA8,N-612-017,Fab1-57,Fab-52,COVOX-278,C548,PD36-5D2,Omi-32,JMB2002
A8	15	(8,13,16,18,19,20,22)	BD-836,UT28K,CV503,Windsor-G32A4,COV2-2196,STE90-C11,P5A-1B8,LY-CoV481,FI-3A,B38,A19-61-1,910-30,10D12-ab1,CV2-1169,B1-182-1,A23-58-1
A9	15	(2,11,12,15,17,18,19,20)	BD-368-2,Zhou-47D1,PDI-93,P2B-2F6,P1D9,Omi-31,NA8,N-612-017,Fab1-57,Fab-52,COVOX-278,DH1041,WRAIR-2173,P2B4,NE12,CV05-163
A10	11	(7,9,11,12,13,16,17,18,19,20,21,22)	PDI-210,S-B8,Beta-29,PR1077,REGN10933,C1A-B3,Beta-22,S2K146,MW07,XGv051,Ab445,P5A-1B6
A11	10	(4,5,6,23)	S304-O1,S304,Liu-10-28,EY6A,S2A4,M8a-6,M8a-3,GH-12,CR3022,C1C-A3,Asarnow-3D11
A12	10	(13,16,18,19)	T6,S5D2,S-E6,RBD-chAb-45,PR953,M31A7,CV07-250,8D3,7F15,7F12,7F0X
A13	10	(13,16,17,18,19,20)	P5C3,P5A-3C12,Hu-Clone6,COVOX-45,WCSL-129,HbnC3-C6,AZD-8895,13G9,THSC20-HVTR26,BD-623,ab4
A14	9	(1,2,3,10,11,12,14,15,17,19,20,22)	G32R7,ab5,PDI-215,Li-D2,COVOX-58,BD-813,6M6,35B5,BD-667,CV07-270
A15	8	(3,11,12,15,16,17,18,19,20)	BG1-24,S2D106,MW05,DH1043,DH1042,Bamlanivimab,553-60,Beta-38,DH1041
A16	8	(8,11,12,13,15,16,17,18,19,20,21,22)	BG7-20,S-B8,PR1077,C144,BD23,S2K146,MW07,Ab188,C051
A17	7	(4,5,6,7)	S2X259,S2A4,M8a-6,M8a-3,GH-12,CR3022,C1C-A3,Asarnow-3D11
A18	7	(4,5,6,7,8,9,13)	COVA1-16,Liu-10-40,Fab2-36,Windsor-G32Q4,VacW-209,M8a-34,Goike-N3-1,ADI-62113
A19	7	(11,13,15,17,18,19,20)	Asarnow-5A6,DH1041,Fab2-43,COVOX-316,Ab496,Ab354,2H2,Omi-9
A20	7	(8,9,11,13,16,17,18,19,20,21)	BD-771,Ab188,COVA2-39,J08,PDI-42,BD-508,BD-503,P2B-1A10
A21	6	(3,12,13,15,17,18,19,20)	P17,553-60,Ab326,Omi-9,Omi-25,Fab-15033-7,Fab-15033
A22	5	(16,17,18,19)	CV2-6264,Beta-54,Ab159,WCSL-119,CR3022-B6,S2H13
A23	5	(4,5,6,7,8,22)	STS165,BD55-1239,S2X35,RBD-chAb-15,Ab-3467,553-15
A24	5	(4,5,6,7,21,22)	DH1047,GW01,BD55-5514,S2X35,RBD-chAb-15,Ab-3467
A25	4	(7,8,21,22)	ADI-55688,3C1,REGN10985,RBD-chAb-25,ADG-20
A26	4	(4,5,6,7,10,22)	MW06,CV2-75,S2X35,RBD-chAb-15,Ab-3467
A27	4	(4,5,7,10,21,22)	K398-22,K288-2,BD55-4637,GW01,BD55-5514
A28	4	(1,2,4,10,11,12,22)	BG10-19,SW186,CV38-142,SP1-77,XG014
A29	3	(4,7,21,22)	Beta-32,REGN10985,RBD-chAb-25,ADG-20
A30	3	(2,4,10,11,21)	ZWC6,C135,BD-821,ZWD12
A31	3	(7,11,20,21,22)	C119,Beta-6,Beta-40,Beta-24
A32	3	(10,11,20,21,22)	Beta-44,Beta-6,Beta-40,Beta-24
A33	3	(3,9,13,14,15,23)	ION-300,WRAIR-2057,S2H97,HSW-1

Supplementary Table 3b

CLUSTER	MEMBERS	ES	LIST (Similarity>=0.85)
N1	13	(4,5,6,7,8,10,21,22)	Xu-Nb12,VHH-72,Nb95,VHH-V,1-2C7,Xu-Nb30,WNb-10,VHH-W,VHH-E,Re9D06,NM1226,Nb34,Hong-7A3,Huo-C1
N2	9	(7,8,9,11,12,13,16,17,18,19,20,21,22)	B01,DL4,WNb-2,Re5D06,NIH-CoVnb-112,MR17,Sb14,Sb16,Nb6,Huo-C5
N3	7	(3,8,11,12,13,15,17,18,19,20)	Nb20,Sota-1C,NB1B11,F6,Nb21,Huo-H3,aRBD-7,aRBD-5
N4	6	(2,11,12,13,15,17,19,20)	H11-H4m,H11-H4,H11-D4,H11-H6,H11-B5,H11-A10,H11
N5	6	(2,3,11,12,14,15,17,18,19,20)	Maeda-P86,Sota-1C,Ty1,K-874A,VHH-G09,Nb17,DL28
N6	6	(2,3,7,8,11,12,13,14,15,17,18,19,20,22)	Hong-8A2,WNb-2,Re5D06,NIH-CoVnb-112,MR17,Sb45,NM1230
N7	5	(4,5,6,7,8,9,22)	VHH-F04,NB1A7,Huo-C1,Nb-2-38,Nb-1-8,Nb-1-25
N8	4	(11,12,13,17,18,19,20)	Wu-Nb22,H11-H6,H11-B5,H11-A10,H11
N9	4	(11,12,13,15,16,17,18,19,20,21,22)	Casasnovas-2-15,Huo-C5,VHH-U,VHH-12,VH-ab6
N10	4	(1,2,3,10,11,12,14,15,17,19,20,21)	Bn03-n3113v,VHH-G09,Nb17,DL28,N3113

Supplementary Tables 3a and 3b. **List of clusters based on the similarity of ES.** **a** 33 Antibody clusters from A1 to A33. **b** 10 Antibody clusters from N1 to N10. Each cluster is grouped as a specific ES combination with a similarity of 0.85 or higher for the members. The similarity of ES between different clusters is less than 0.85.

Supplementary Table 4

a

Name	PDB	Sim	ES=(**2**,3,10,11,12,15,17,19)	Multi-contacts
**Antibody**				
**G32R7**	**7n64**	0.80	(1,**2**,3,10,11,12,14,15,17,19,20,22)	(2,**7**,4,1,3,2,1,3,4,1,1,1)
Li-D2	7xmz	0.78	(1,**2**,3,11,12,14,15,17,19,20)	(2,**7**,3,2,2,1,2,1,1,1)
6M6	7wk0	0.78	(1,**2**,3,11,12,14,15,17,19,20)	(3,**7**,4,3,3,1,4,3,1,1)
PDI-215	7mzm	0.78	(1,**2**,3,11,12,14,15,17,19,20)	(2,**6**,7,4,2,1,3,1,1,1)
COVOX-58	7qny	0.78	(1,**2**,3,11,12,14,15,17,19,20)	(2,**6**,5,2,2,1,3,3,1,1)
35B5	7e9p	0.78	(1,**2**,3,11,12,14,15,17,19,20)	(2,**6**,4,2,2,1,2,4,1,1)
ab5	7e3b	0.89	(**2**,3,10,11,12,14,15,17,19,22)	(**5**,3,3,3,3,1,3,3,1,1)
BD-813	7ey0	0.78	(1,**2**,3,11,12,14,15,17,19,20)	(2,**5**,5,1,2,1,2,4,1,1)
**Omi-32**	**7zfe**	0.80	(**2**,3,11,12,15,19,20)	(**5**,2,4,2,1,1,1)
**Nanobody**				
**N3113**	**7vnb**	0.82	(1,**2**,3,11,12,15,17,19,20)	(1,**6**,4,2,2,4,2,1,3)
Bn03-n3113v	7whi	0.80	(1,**2**,3,10,11,12,14,15,17,19,20,21)	(1,**5**,4,2,5,2,1,3,1,1,2,1)
DL28	7f5h	0.82	(**2**,3,11,12,14,15,17,19,20)	(**5**,6,2,3,1,5,4,1,1)
N3113-1	7vne	0.80	(**2**,3,11,12,15,19,20)	(**5**,2,1,2,2,1,3)

b

Name	PDB	Sim	ES=(17,**18**,19)	Multi-contacts
**Antibody**				
P5A-3C12	7d0b	0.75	(13,17,**18**,19,20)	(1,2,**3**,2,1)
**COVOX-45**	**7bel**	0.75	(13,17,**18**,19,20)	(2,1,**3**,3,1)
THSC20-HVTR26	7z0x	0.75	(13,16,17,**18**,19)	(1,4,2,**3**,3)
BD-623	7e7y	0.75	(13,16,17,**18**,19)	(1,5,2,**3**,3)
ab4	7e39	0.75	(13,16,17,**18**,19)	(1,3,1,**3**,1)
Beta-26	7q9j	0.75	(12,15,17,**18**,19)	(2,1,4,**3**,1)
CV2-6264	7qf1	0.86	(16,17,**18**,19)	(3,2,**3**,1)
Beta-54	7ps6	0.86	(16,17,**18**,19)	(5,2,**3**,2)
CR3022-B6	7kzb	1.00	(17,**18**,19)	(1,**3**,2)
**Nanobody**				
**Nb-2-67**	**8cya**	0.67	(13,16,17,**18**,19,20)	(2,4,2,**3**,3,1)

Supplementary Table 4a and 4b. **a** A cluster of antibodies and nanobodies that have multiple contacts with ES2 (R346). The list of Abs and Nbs with a similarity (Sim) to ES=(2,3,10,11,12,15,17,19) are sorted by the multiplicity at ES2. The numbers in brackest of "Multi-contacts" are the multiplicity of contacts corresponding to each ES site number. **b** A cluster of antibodies and nanobodies with a similarity (Sim) to ES=(17,18,19) that have multiple contacts with ES18 (F486). A major distinct newly effective mutation of XBB.1.5 is F486P (ES18).

Supplementary Fig. 4

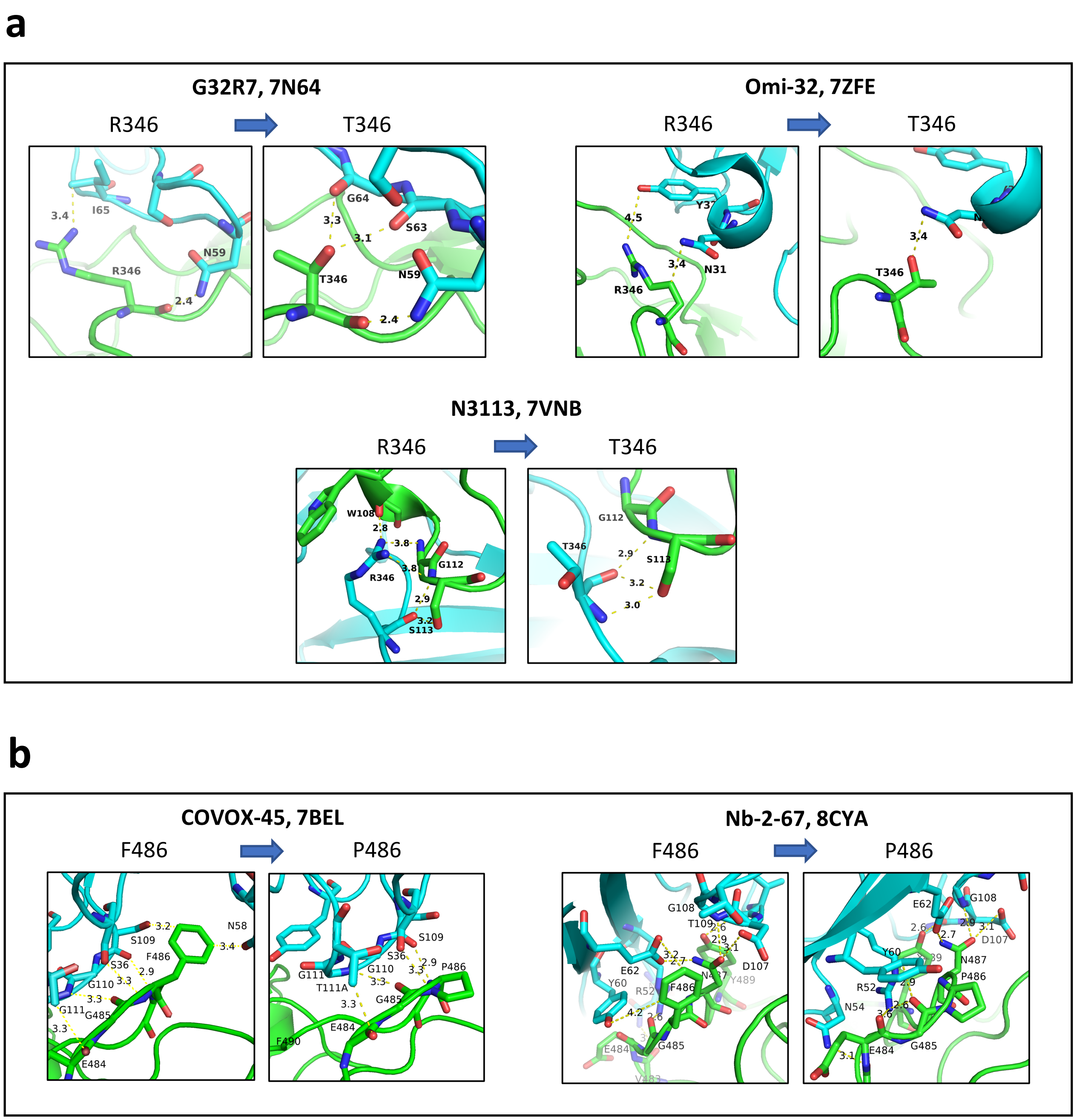

Supplementary Fig. 4a and 4b **a** Illustration of the location of the escape mutation (R346T) at ES2 for two antibodies (G32R7 and Omi-32), one nanobody (N3113). Omi-32 is the latest structure that is against sub-variants of BA.1 and BA.2 [3,4]. **b** Illustration of the models that may prevent the escape mutation. COVOX-45: there are multiple contacts from main-chain of CDR3 to the loop of F486, although F486 mutates to P486. The P486 would not escape; Nb-2-67: although F486 mutated to P486, the mainchain contacts to R52 and Y60 do not change. There are also multiple hydrogen bonds to keep the E18 loop in contact.

Supplementary Fig. 5

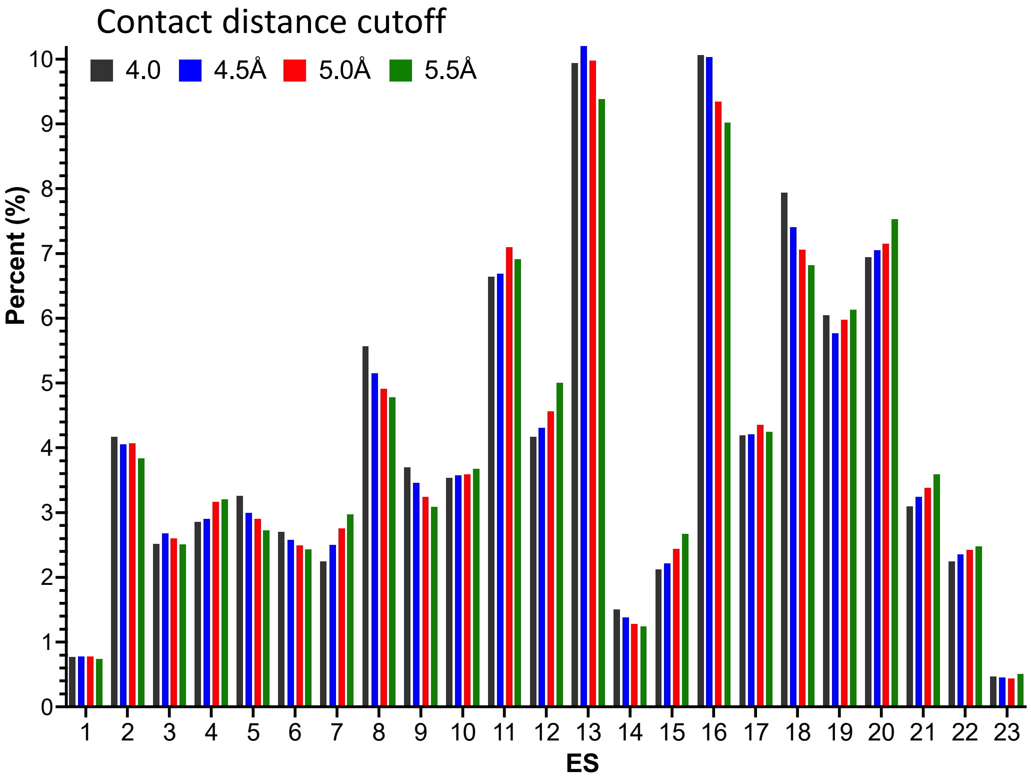

ES	RANGE	RBD	Distance 4.0 Hits	Distance 4.0 (%)	Distance 4.5 Hits	Distance 4.5 (%)	Distance 5.0 Hits	Distance 5.0 (%)	Distance 5.5 Hits	Distance 5.5 (%)
1	339-341	GEV	33	0.77	39	0.78	44	0.78	46	0.74
2	343-349	NTRFAS	179	4.17	203	4.06	229	4.07	239	3.84
3	351-357	YAWNRKR	108	2.52	134	2.68	146	2.60	156	2.51
4	368-374	LYNSASF	123	2.86	145	2.90	178	3.17	199	3.20
5	375-380	STFKCY	140	3.26	150	3.00	163	2.90	170	2.73
6	381-386	GVSPTK	116	2.70	129	2.58	140	2.49	151	2.43
7	403-409	RGDEVRQ	96	2.24	125	2.50	155	2.76	185	2.97
8	411-417	APGQTGK	239	5.57	258	5.15	276	4.91	297	4.78
9	420-428	DYNYKLPDD	159	3.70	173	3.46	182	3.24	192	3.09
10	437-443	NSNNLDS	152	3.54	179	3.58	202	3.59	229	3.68
11	444-449	KVGGNY	285	6.64	335	6.69	399	7.10	430	6.91
12	450-454	NYLYR	179	4.17	216	4.31	257	4.57	311	5.00
13	455-460	LFRKSN	427	9.94	521	10.41	561	9.98	584	9.39
14	462-467	KPFERD	65	1.51	69	1.38	72	1.28	77	1.24
15	468-472	ISTEI	91	2.12	111	2.22	137	2.44	166	2.67
16	473-479	YQAGSTP	432	10.06	502	10.03	525	9.34	561	9.02
17	481-484	NGVE	180	4.19	211	4.21	245	4.36	264	4.25
18	485-487	GFN	341	7.94	371	7.41	397	7.06	424	6.82
19	488-491	CYFP	260	6.05	289	5.77	336	5.98	381	6.13
20	492-496	LQSYG	298	6.94	353	7.05	402	7.15	468	7.53
21	497-502	FQPTNG	133	3.10	162	3.24	190	3.38	223	3.59
22	503-509	VGYQPYR	96	2.24	118	2.36	136	2.42	154	2.48
23	516-520	ELLHA	20	0.47	23	0.46	25	0.44	32	0.51

Supplementary Fig. 5 The distribution of contacts in percentage for each ES area with respect to the distance cutoff at 4.0 (gray), 4.5 (blue), 5.0 (red) and 5.5 (green) in Å. The represent the fraction of the H-chains of 340 antibodies.

Supplementary Fig. 6

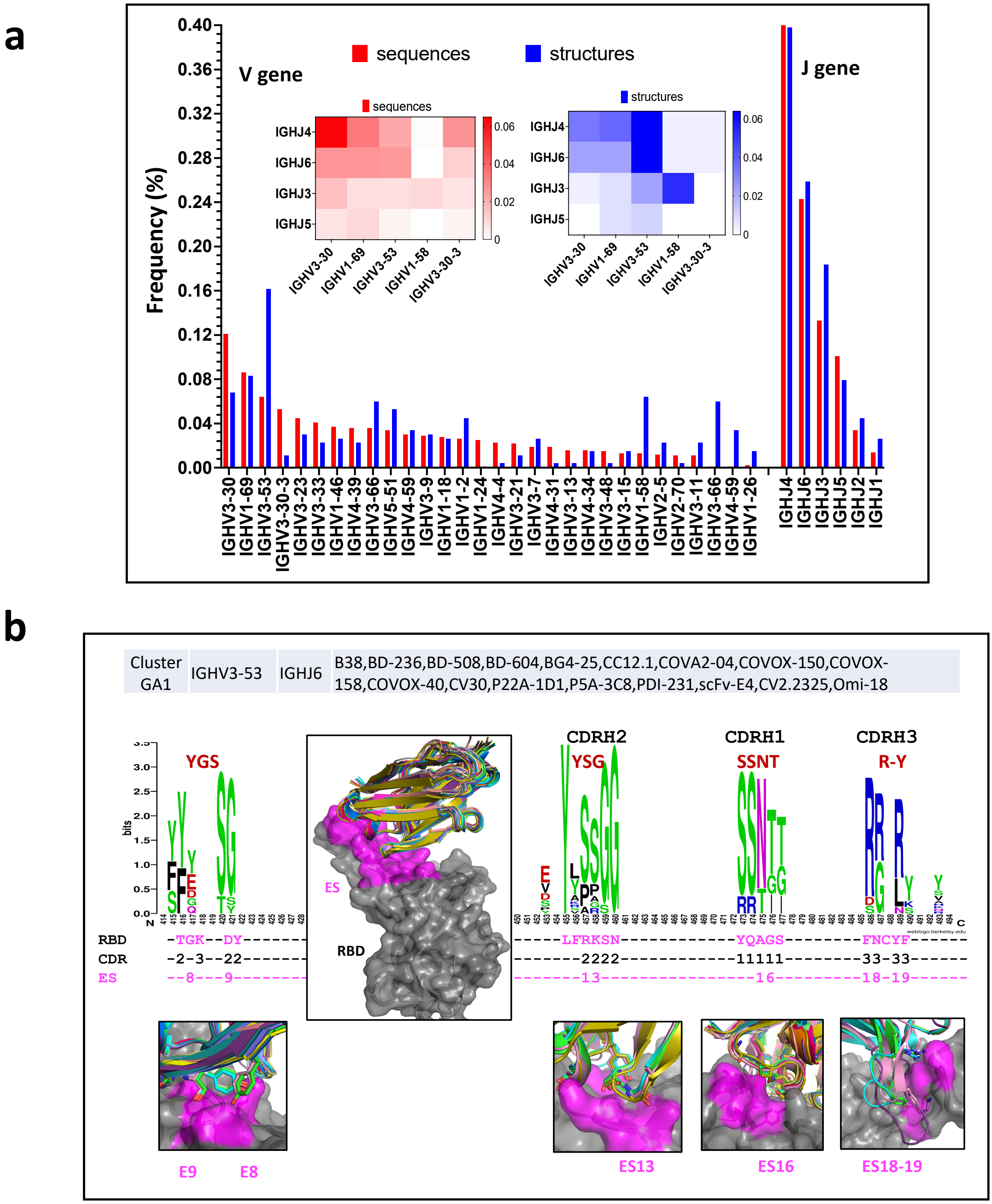

Supplementary Fig. 6. a The usage of V(D)J gene and combinations of antibody H-chain. The gene information is taken from Cov-AbDab with a total of 6316 antibody sequences, unique HV gene ids are 118 and unique HJ gene ids are 8. Only 266 presentative structures are found among of these mAbs. A frequency of sequences greater than 1% is plotted as descending order (red). The frequency of structures is plotted side by side (blue). Insertion heat maps presented the VJ combination (red for sequences, blue for structures). **b** A cluster GA1 based on the gene similarity (IGHV3-53+IGHJ6) is presented. The ES combination is (8,9,13,16,18,19), and the binding motifs are SSNT for CDRH1, YSG for CDRH2, and RY for CDRH3, which is similar to the Cluster A1S1 (Figure 5c). Insertion figure shows superimposed structures of the GA1 cluster.

Supplementary Fig. 7

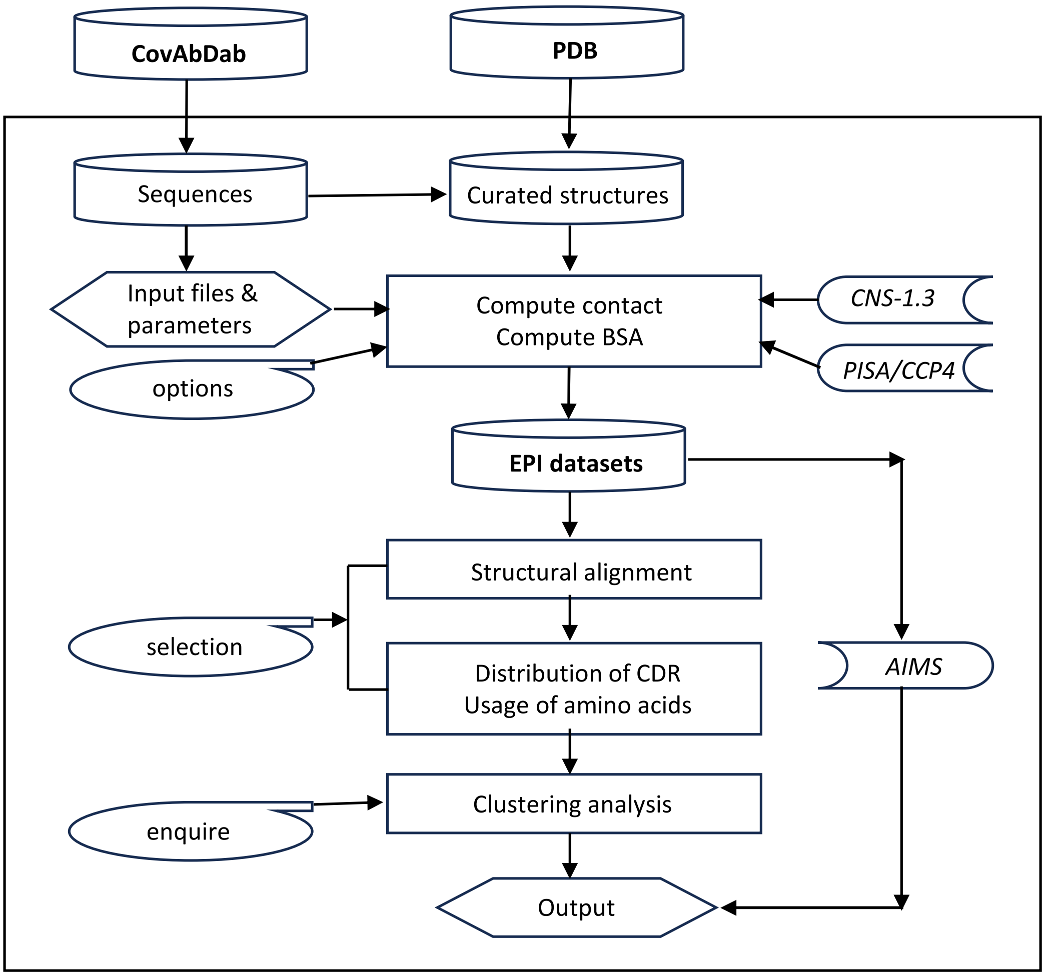

Supplementary Fig. 7 A flowchart of EPI software package. The package utilizes the 3rd party software: *CNS-1.3, PISA/CCP4*, and *AIMS.*

References for Supplementary Figures and Tables

1 Barnes, C. O. *et al.* Structures of Human Antibodies Bound to SARS-CoV-2 Spike Reveal Common Epitopes and Recurrent Features of Antibodies. *Cell* **182**, 828-842 e816 (2020). https://doi.org:10.1016/j.cell.2020.06.025

2 Crooks, G. E., Hon, G., Chandonia, J. M. & Brenner, S. E. WebLogo: A sequence logo generator. *Genome Research* **14**, 1188-1190 (2004).

3 Nutalai, R. *et al.* Potent cross-reactive antibodies following Omicron breakthrough in vaccinees. *Cell* **185**, 2116-2131 e2118 (2022). https://doi.org:10.1016/j.cell.2022.05.014

4 McCallum, M. *et al.* Structural basis of SARS-CoV-2 Omicron immune evasion and receptor engagement. *Science* **375**, 864-868 (2022). https://doi.org:10.1126/science.abn8652
