# Supplementary material for: SARS-CoV-2 antibodies recognize 23 distinct epitopic sites on the receptor binding domain
Source: Commun Biol. 2023 Sep 19;6:953. doi: 10.1038/s42003-023-05332-w (PMC10509263; doi:10.1038/s42003-023-05332-w)
Supplement: Supplementary file 3 — Description of Additional Supplementary Files [file 42003_2023_5332_MOESM3_ESM.pdf]

Description of Additional Supplementary Files

File name: Supplementary Data 1
Description: Each of the Ab structures taken from the PDB is listed by Name, PDB ID, epitope chain, and paratope chain designation. (see Methods). Whether the structure was derived from Ab/RBD (R) or Ab/spike (S) and the experimental method (X-ray (X) or cryo-EM (E) is also indicated. Resolution of the structure (in Å) and buried surface area (BSA in Å2) of the H or L chain and H+L chains (for Ab) to the RBD interface (see Methods) for the indicated Ab or Nb is given. The designation of the epitopic sites (ES) in the H chain or Nb interface is also given. Columns of "Variants" and a list of "Mutations (RBD)" are listed from the structure (PDB) file.

File name: Supplementary Data 2
Description: Each of the Nb structures taken from the PDB is listed by Name, PDB ID, epitope chain, and paratope chain designation. (see Methods). Whether the structure was derived from Ab/RBD (R) or Ab/spike (S) and the experimental method (X-ray (X) or cryo-EM (E) is also indicated. Resolution of the structure (in Å) and buried surface area (BSA in Å2) of the H or L chain and H+L chains (for Ab) to the RBD interface (see Methods) for the indicated Ab or Nb is given. The designation of the epitopic sites (ES) in the H chain or Nb interface is also given. Columns of "Variants" and a list of "Mutations (RBD)" are listed from the structure (PDB) file.

File name: Supplementary Movie 1
Description: Each ES is illustrated with the secondary structure of RBD.

File name: Supplementary Movie 2
Description: Each ES surface area or footprint is illustrated by a color map of the RBD surface.
